# Establishment of porcine and monkey colonic organoids for drug toxicity study

**DOI:** 10.1186/s13619-021-00094-4

**Published:** 2021-10-02

**Authors:** Haonan Li, Yalong Wang, Mengxian Zhang, Hong Wang, Along Cui, Jianguo Zhao, Weizhi Ji, Ye-Guang Chen

**Affiliations:** 1 The State Key Laboratory of Membrane Biology, Tsinghua-Peking Center for Life Sciences, School of Life Sciences, Tsinghua University, Beijing, 100084 China; 2 State Key Laboratory of Primate Biomedical Research, Institute of Primate Translational Medicine, Kunming University of Science and Technology, Kunming, 650500 Yunnan China; 3 School of Life Sciences, University of Science and Technology of China, Hefei, 230027 China; 4 State Key Laboratory of Stem Cell and Reproductive Biology, Institute of Zoology, Chinese Academy of Sciences, Beijing, 100101 China; 5 University of Chinese Academy of Sciences, Beijing, 100049 China; 6 Max-Planck Center for Tissue Stem Cell Research and Regenerative Medicine, Guangzhou Regenerative Medicine and Health Guangdong Laboratory, Guangzhou, 510700 China

**Keywords:** Pig, Monkey, Colonic organoids, Culture, Drug toxicity

## Abstract

Pig and monkey are widely used models for exploration of human diseases and evaluation of drug efficiency and toxicity, but high cost limits their uses. Organoids have been shown to be promising models for drug test as they reasonably preserve tissue structure and functions. However, colonic organoids of pig and monkey are not yet established. Here, we report a culture medium to support the growth of porcine and monkey colonic organoids. Wnt signaling and PGE2 are important for long-term expansion of the organoids, and their withdrawal results in lineage differentiation to mature cells. Furthermore, we observe that porcine colonic organoids are closer to human colonic organoids in terms of drug toxicity response. Successful establishment of porcine and monkey colonic organoids would facilitate the mechanistic investigation of the homeostatic regulation of the intestine of these animals and is useful for drug development and toxicity studies.

**Supplementary Information:**

The online version contains supplementary material available at 10.1186/s13619-021-00094-4.

## Background

Rodents, especially mice, have been widely used in biomedical research. However, mice do not exhibit the similar pathological features of human gastrointestinal disease. For instance, Adenomatous polyposis coli (APC) mutation-associated polyps are usually found in the colorectum in human, while APC-induced polyp formation occurs preferably in the small intestine in mice (Boivin et al. 2003; Flisikowska et al. 2012; Moser et al. 1990). Pig and cynomolgus monkey are regarded as ideal animal models to investigate the human gastrointestinal function and disease based on the similarity of genomic sequence, anatomic morphology and drug metabolism with human beings (Bray et al. 2018; Cibelli et al. 2013; Deglaire and Moughan 2012; Kararli 1995; Patterson et al. 2008; Ziegler et al. 2016). Multiple gastrointestinal disease models are established in pigs, such as short-bowel syndrome (Pereira-Fantini et al. 2011; Vegge et al. 2013) and colorectal cancer (Flisikowska et al. 2012). Accordingly, pigs have been approved for pharmaceutical testing by United States Food and Drug Administration (Gonzalez et al. 2015).

In the last decade, organoids have been demonstrated to be a great model for disease study, drug test and regeneration medicine. Organoids derived from adult stem cells possess the capability to maintain self-renewal while being able to differentiate into functional cell types, mimic the three-dimension (3D) structures and retain the functions of the origin tissues (Schutgens and Clevers 2020; Zhang et al. 2020). In 2009, Clevers and colleagues developed the first meaningful mouse intestinal organoids embedded in Matrigel with the culture medium supplemented with epidermal growth factor (EGF), Noggin and R-spondin (Sato et al. 2009). Subsequently, mouse colonic organoids and human intestinal organoids were also established successfully (Fujii et al. 2018; Jung et al. 2011; Sato et al. 2011). By far, canine, bovine, porcine, bat, feline, chicken intestinal organoids have been cultured successfully (Chandra et al. 2019; Derricott et al. 2019; Gonzalez et al. 2013; Khalil et al. 2016; Kramer et al. 2020; Powell and Behnke 2017; Zhou et al. 2020). However, in our best knowledge, the porcine and monkey colonic organoid models are not reported. Here, we report the establishment of the organoids derived from the colon of pig and monkey. Using these organoids, we carried out drug toxicity studies and found that porcine and human colonic organoids exhibited similar toxicity response. These organoid models provide a useful platform to expand the species investigation and drug toxicity studies.

## Results

### Establishment of porcine and monkey colonic organoids

**Fig. 1:**
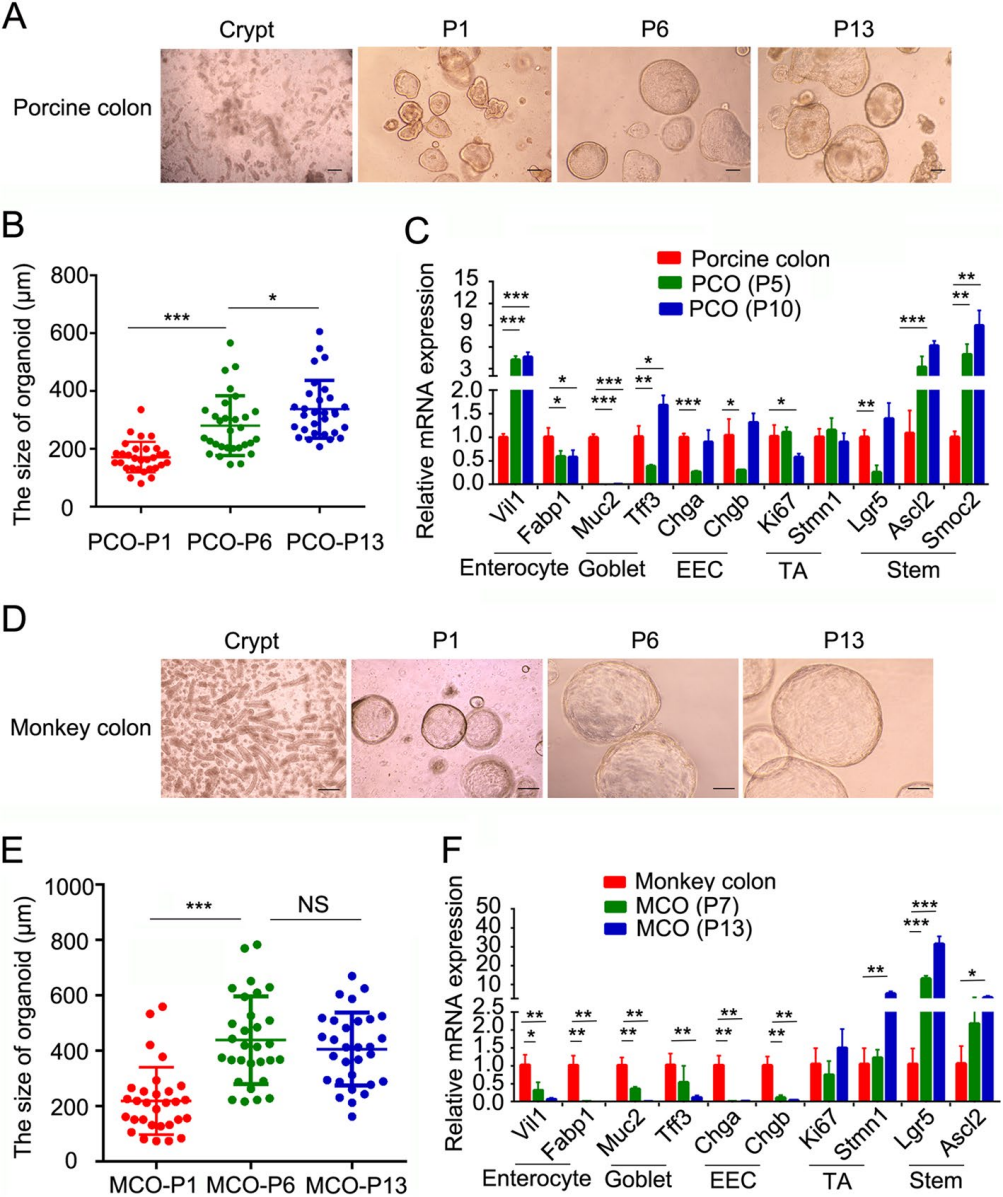
Establishment of porcine and monkey colonic organoids. **A**, **D** Representative images of organoid growth of porcine colon (**A**) and monkey colon (**D**) in expansion medium from different passages. **B**, **E** Quantitation of the size of porcine colonic organoids (PCOs) (**B**) and monkey colonic organoids (MCOs) (**E**) in different passages. **C**, **F** Expression of cell marker genes was examined with q-PCR in primary colon tissue and organoids from different passages in pig (**C**) and monkey (**F**). Scale bars, 100 μm. *p < 0.05, ** *p* < 0.01, ***p < 0.001. Data are displayed as the mean ± SD by one-way ANOVA (**B** and **E**) and Student’s t-test (**C** and **F**). 30 organoids were calculated for the size in **B** and **E**. Three independent experiments were performed in **C** and **F**

Fresh crypts from adult porcine colon were harvested and embedded into Matrigel and cultured with expansion medium (EM) based on previously reported human colonic organoid medium (Jung et al. 2011). The sphere organoids were observed without budding structures after 1 week (Fig. 1A), and these organoids were maintained for at least 16 passages about 3 months. The size of porcine colonic organoids (PCOs) was increased after several passages, indicating fast proliferation in EM (Fig. 1B). The expression of trans-amplifying (TA) cell markers was similar between PCOs and the colon tissue. Meanwhile, the expression of stem cell marker genes (especially for *Ascl2* and *Smoc2*) was significantly increased in PCOs compared to colon tissue, indicating the enrichment of stem cells in PCOs (Fig. 1C) (Munoz et al. 2012; van der Flier et al. 2009). The marker genes of mature cell types including enterocytes (marked by *Fabp1*), goblet cells (marked by *Muc2* and *Tff3*), enteroendocrine cells (EECs) (marked by *Chga* and *Chgb*) had decreased expression in early passages of PCOs compared to the colon tissue, and their expression recovered (especially for EEC marker genes) in late passages of PCOs except *Muc2* (Fig. 1C). *Vil1* (villin1) mainly marks enterocytes, but is also expressed in other immature epithelial cells in the intestine (el Marjou et al. 2004). The expression of *Vil1* was obviously increased in PCOs, confirming the epithelial identity of PCOs.

The similar culture procedure was conducted to culture organoids from adult cynomolgus monkey colon. EM also supported the growth of monkey colonic organoids (MCOs) (Fig. 1D), which could be maintained for at least 15 passages about 75 days. Fast proliferation was detected as the organoid size was significantly increased before passage 6, and then the organoid size was stable (Fig. 1E). The expression of the maker genes for various cell types was compared between MCOs and primary tissues. The expression of enterocytes, goblet cells marker genes was significantly reduced in organoids compared to colon tissue, and EECs marker *Chga* and *Chgb* were rarely detected in the organoids (Fig. 1F). However, the stem cells and TA cells markers exhibited increased expression compared to primary monkey colon tissue. Together, EM could support porcine and monkey colonic organoid growth for long time and maintain the proliferation ability.

### Colonic organoids undergo differentiation in the differentiation medium

**Fig. 2:**
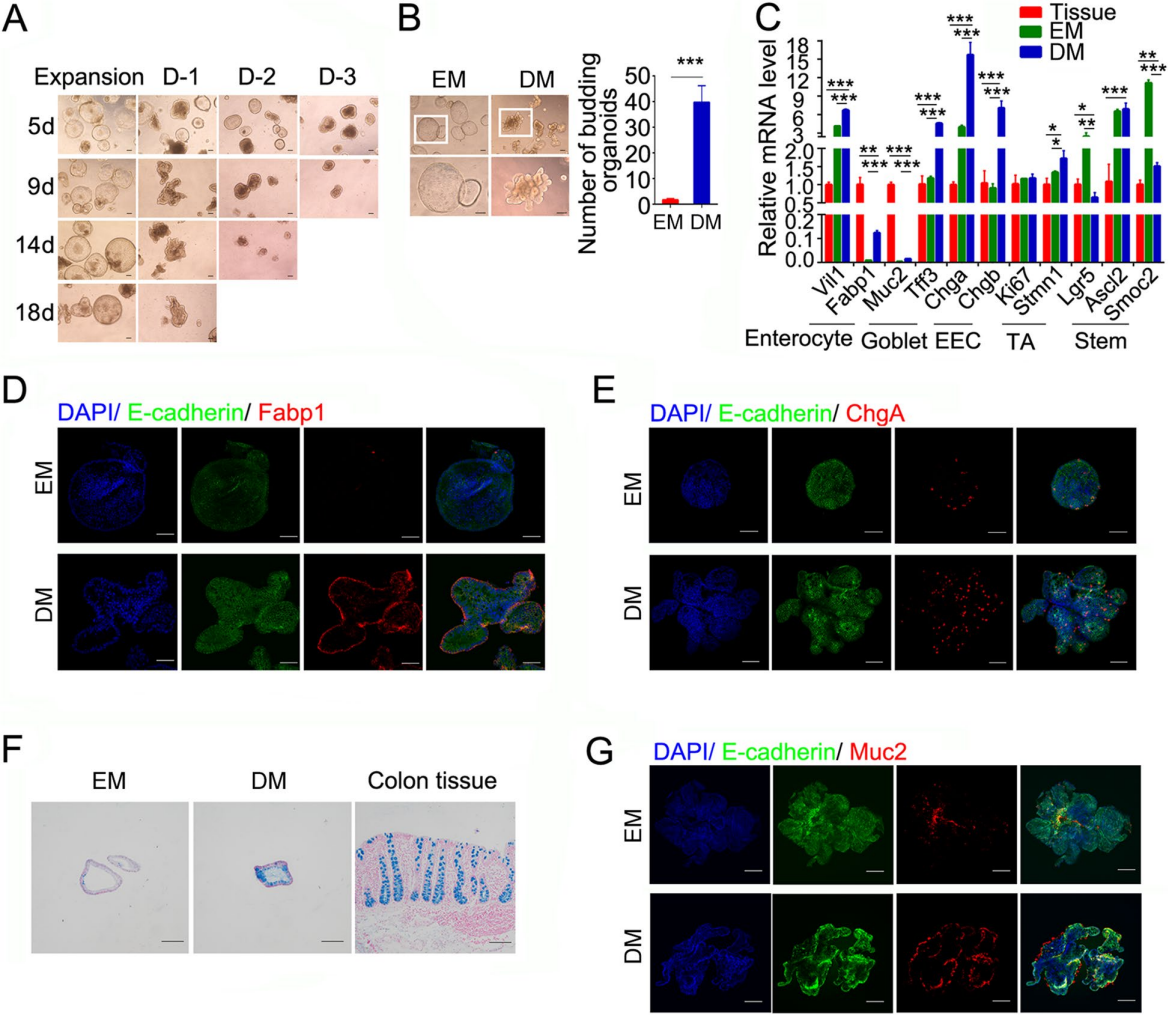
Mature lineages of porcine colonic organoids are induced in differentiation medium. **A** Representative images of porcine colonic organoids grown in different differentiation media. Differentiation medium-1 (D-1): withdrawal of Wnt3a-CM and PGE2 from expansion medium with 2.5 μM CHIR-99021; Differentiation medium-2 (D-2): withdrawal of Wnt3a-CM, PGE2 and CHIR-99021 from expansion medium; Differentiation medium-3 (D-3): withdrawal of Wnt3a-CM, PGE2, CHIR-99021, nicotinamide and SB202190 from expansion medium. **B** Representative bright-field images and quantitation of the budding numbers of organoids in porcine colonic organoids cultured in expansion or differentiation medium for 7 days. White box depicts higher magnification below. **C** Expression of cell marker genes in proliferating (EM), differentiated (DM) porcine colonic organoids (passage 15) and tissue. **D**-**G** Fabp1 staining (**D**), ChgA staining (**E**), Alcian blue staining (**F**), Muc2 staining (**G**) in porcine colonic organoids cultured in expansion or differentiation medium for 7 days. Scale bars, 100 μm. *p < 0.05, ** *p* < 0.01, ***p < 0.001 analyzed by Student’s t-test. Data are shown as mean ± SD (*n* = 3 independent experiments)

Both PCOs and MCOs were in a high proliferation state with less differentiated mature cell types in EM. The homostatic balance of proliferating vs. differentiation cells in the intestine epithelium tissue is achieved with combinatory effect of various niche factors (Zhang et al. 2020; Zhu et al. 2021). To recapitulate the physiological cell constitution, we developed three differentiation media: (1) Differentiation-1 (D-1): withdrawal of Wnt3a-conditional medium (Wnt3a-CM) and PGE2 from EM and the concentration of CHIR-99021 was decreased to 2.5 μM; (2) D-2: withdrawal of Wnt3a-CM, PGE2 and CHIR-99021; (3) D-3: withdrawal of Wnt3a-CM, PGE2, CHIR-99021, nicotinamide and SB202190. Then PCOs grown in EM were transferred into these differentiation media. Solid or budding organoids were found in D-1, -2, -3 media at day 5 (Fig. 2A). However, organoids started to die in D-2 and -3 media in 1 to 2 weeks, while the organoids in D-1 could be maintained for at least 3 weeks. Since Wnt signaling plays a critical role in intestinal stem cell self-renewal (Barker 2014; Qi and Chen 2015; Sato and Clevers 2013; Yin et al. 2014), reducing Wnt signaling in differentiation media would lead to decreased organoid proliferation and cell death in long-term culture. Therefore, D-1 medium (DM) was used for the subsequent studies.

Compared to the PCOs growing in EM, morphological changes were observed in DM from sphere to budding organoids (Fig. 2A-B). The expression of mature cell types markers (including *Vil1*, *Fabp1* for enterocytes; *Muc2*, *Tff3* for goblet cells; *Chga*, *Chgb* for EECs) was significantly increased in DM compared to EM (Fig. 2C). However, *Fabp1* and *Muc2* expression remained low even in the PCOs cultured in DM compared to tissue, indicating an incomplete differentiation process in PCOs. The expression of TA cell markers (*Ki67* and *Stmn1*) was not changed or slightly increased, and the stem cell markers (*Lgr5* and *Smoc2*) in EM returned to the levels close to ones in the tissue. Reducing Wnt signaling in DM could directly counts for the decreased *Lgr5* expression in PCOs. To further verify mature cell types in the DM-cultured PCOs, immunofluorescence staining for enterocytes, EECs and goblet cells were performed. As shown in Fig. 2D, Fabp1^+^ enterocytes were detected in the DM-cultured organoids, but were barely observed in the EM-culture organoids. Similarly, ChgA^+^ EECs were significantly increased with DM culture (Fig. 2E). Furthermore, more Muc2^+^ goblet cells were observed in the DM-cultured organoids, as shown by immunofluorescence and Alcian blue staining (Fig. 2F-G). These data demonstrate that differentiation was promoted in DM-cultured PCOs, which can better resemble the composition of the colonic epithelium.

**Fig. 3:**
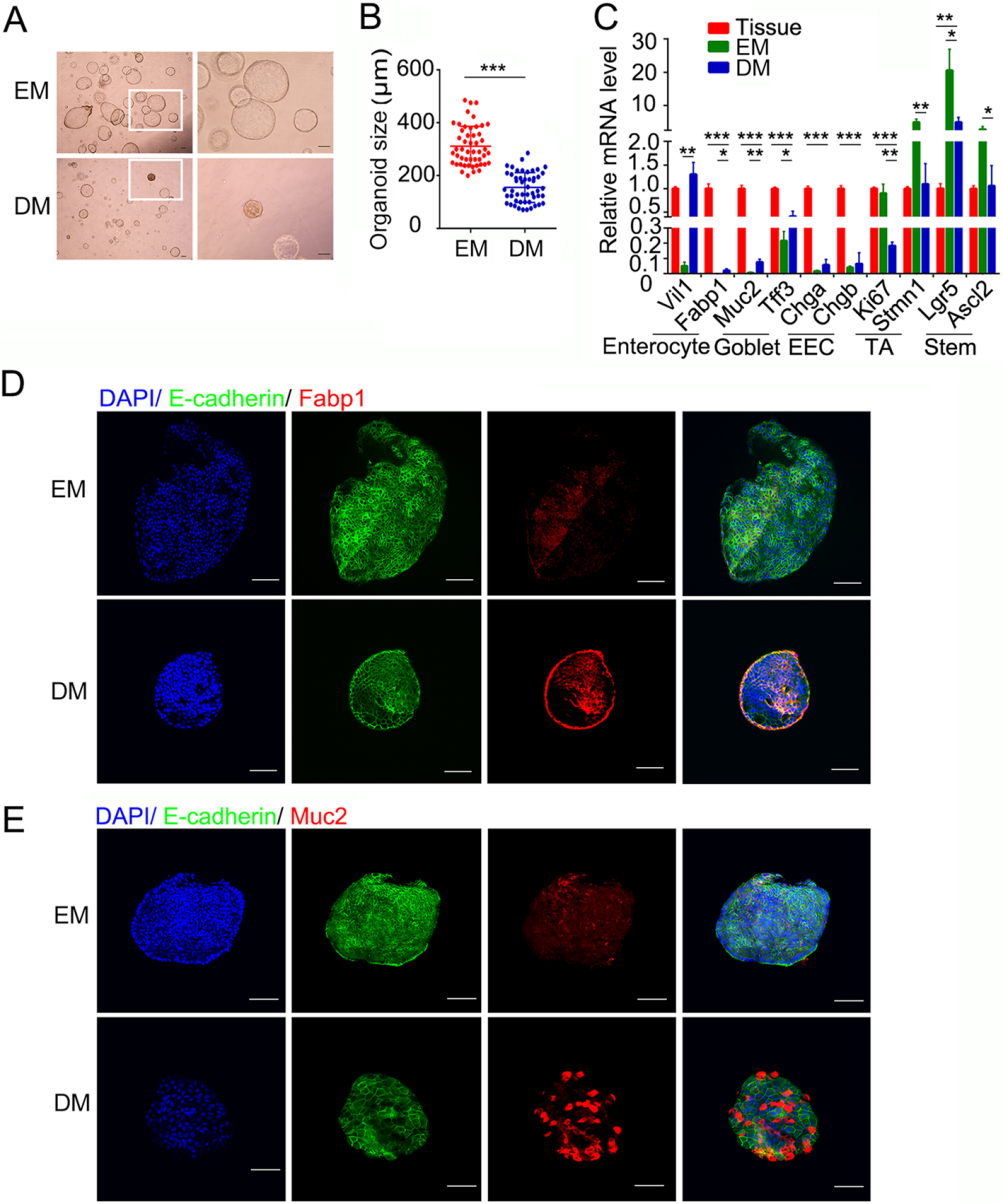
Mature lineages of monkey colonic organoids are induced in differentiation medium. **A**, **B** Representative images (**A**) and quantitation of organoid size (**B**) of monkey colonic organoids cultivated in expansion or differentiation medium for 7 days. White box depicts higher magnification below. **C** Expression of cell marker genes in proliferating (EM), differentiated (DM) monkey colonic organoids (passage 15) and tissue. **D**, **E** Fabp1 staining (**D**), Muc2 staining (**E**) of monkey colonic organoids cultured with expansion or differentiation medium for 7 days. Scale bars, 100 μm. *p < 0.05, ** *p* < 0.01, ***p < 0.001 analyzed by Student’s t-test. Data are shown as mean ± SD (*n* = 3 independent experiments)

To obtain more differentiated mature cells in MCOs, the organoids cultured in EM were transferred into DM for 1 week, and the size of sphere organoids was found to be smaller than the organoids in EM with the appearance of solid organoids (Fig. 3A-B). Accordingly, the expression of TA cells and stem cells markers was significantly decreased to the levels close to the tissue in DM-cultured MCOs, whereas some of differentiated cells markers *Vil1* (enterocytes) and *Tff3* (goblet cells) were upregulated compared to EM (Fig. 3C). Other differentiation markers were slightly increased in the DM-cultured MCOs compared to EM, but still lower than ones in the tissue, suggesting insufficient differentiation process in MCOs. Immunofluorescence staining further confirmed increased Fabp1^+^ enterocytes and Muc2^+^ goblet cells in the DM-cultured MCOs (Fig. 3D-E). These results together indicate that this differentiation medium promotes the differentiation of enterocytes and goblet cells, but not very effective on EECs differentiation, which may suggest the species difference in cell differentiation regulation.

### Porcine colonic organoids are a promising model for drug sensitivity test

**Fig. 4:**
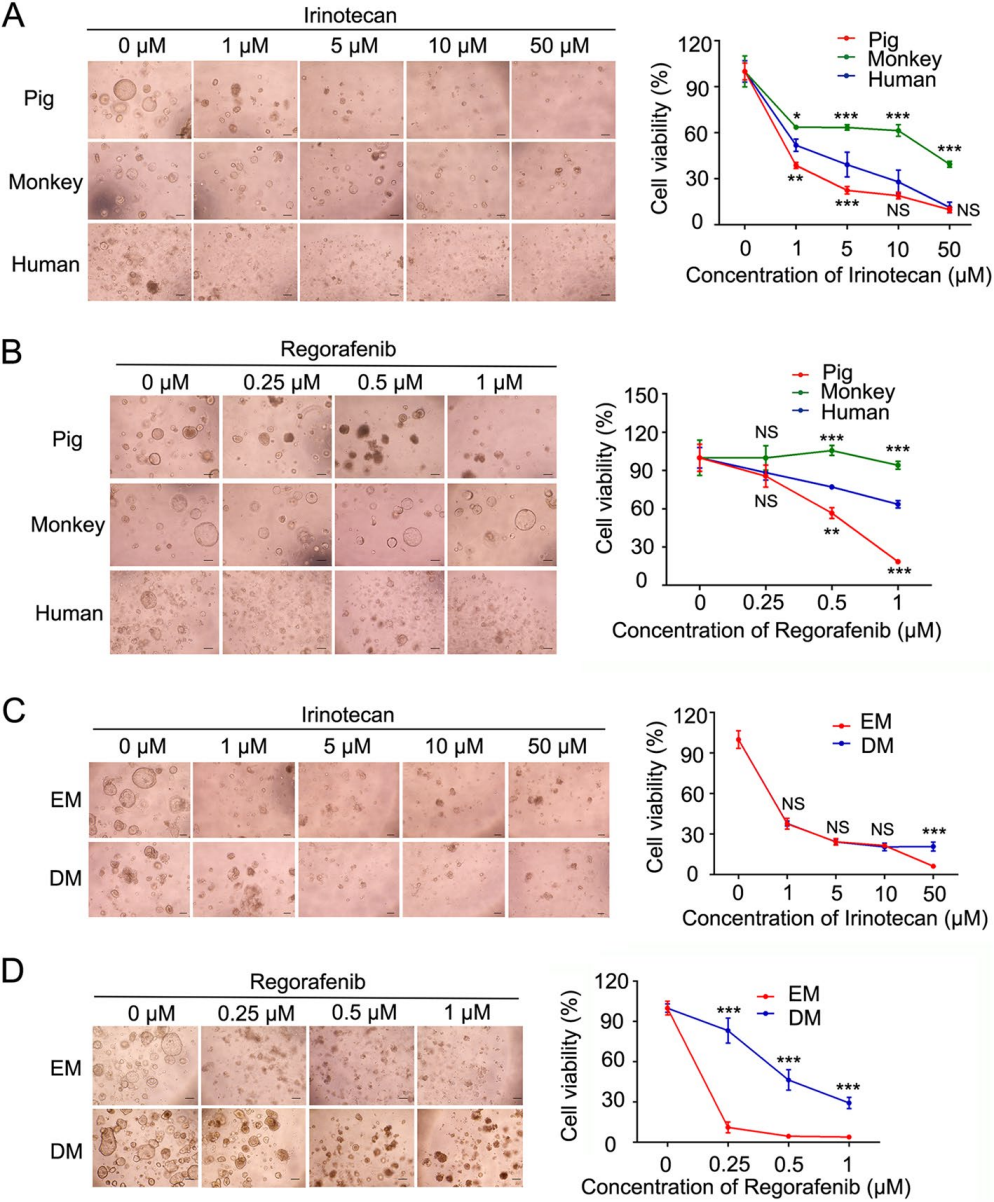
Porcine colonic organoids are more sensitive to drug toxicity. **A**, **B** Representative bright-field images (left) and quantitation of cell viability (right) of porcine, monkey and human colonic organoids treated with different concentrations of irinotecan (**A**) or regorafenib (**B**) for 4 days. Data are shown as mean ± SD (*n* = 3 independent experiments). **C, D** Representative bright-field images (left) and quantitation of cell viability (right) of PCOs in the expansion or differentiation medium treated with irinotecan (**C**) or regorafenib (**D**) for 4 days. Data are shown as mean ± SD by two-way ANOVA (*n* = 3 independent experiments). The significant difference was compared to human colonic organoids. *p < 0.05, ** *p* < 0.01, ***p < 0.001

Organoids are powerful tools to investigate the drug metabolism and toxicity (Burtin et al. 2020; Derricott et al. 2019). Most drug toxicity studies in organoids are carried out with the ones derived from mouse and human (Morizane et al. 2015; Park et al. 2019). Here, we applied porcine, monkey and human colonic organoids to test the toxicity response of anti-cancer drugs. Three types of organoids were seeded into 96 well plates in DM. Then, two clinically used anti-colon cancer drugs irinotecan (a DNA topoisomerase inhibitor) and regorafenib (a multi-targeted receptor tyrosine kinase inhibitor) were separately added into the organoids with various concentrations. After 4 days, the organoid growth and cell viability were examined. The organoid size and numbers were significantly reduced in porcine and human organoids treated with 1 μM irinotecan. Inhibition on cell viability of human and porcine organoids was apparent at 5 μM, and there were rare viable organoids in porcine and human organoids treated with 50 μM irinotecan (Fig. 4A). However, monkey organoids showed the resistance to irinotecan treatment: even in 50 μM irinotecan, about 40% of the monkey organoids still survived. Regorafenib caused 80% cell death in porcine organoids in 1 μM, while monkey organoids were also more resistant to regorafenib (Fig. 4B). These data indicate that PCOs are more sensitive to these drugs than MCOs, and are closer to human colonic organoids.

To investigate the effect of culture conditions on drug sensitivity, we further compared the sensitivity of PCOs in EM or DM for 4 days to irinotecan or regorafenib. As shown in the following Fig. 4C and D, PCOs showed a similar toxicity response to irinotecan in two culture media. However, PCOs had different responses to regorafenib, and the differentiated PCOs were less sensitive, suggesting that highly proliferating cells are more sensitive to some drugs.

## Discussion

In this study, we have successfully established porcine and monkey colonic organoid culture, and these organoids possessed the in vivo cell composition. Our results showed that reduced Wnt signaling and withdrawal of PGE2 induced lineage differentiation. However, when cultured in the differentiation medium for a long time, the organoids started to die. Therefore, there is a trade-off to achieve a balance between proliferation and differentiation in intestinal organoids. Withdrawal of Wnt signaling activity is necessary for enterocyte differentiation in human colonic organoids. The differentiation of goblet cells and EECs is promoted after withdrawal of nicotinamide and SB202190 (Sato et al. 2011). In addition, Notch inhibition could further promote goblet cell hyperplasia and cease proliferation (Otsuka et al. 2010; Sato et al. 2011). These reports provide clues to achieve balanced differentiation. In our differentiation system, D-1 could not only promote mature cell differentiation, but also maintain organoid growth for weeks. It provides a useful platform for functional studies, such as drug toxicity test. However, there is still a gap between differentiated organoids and tissue. D-2 and D-3 may have greater differentiated ability, while obvious growth inhibition and cell death limit their application. A more applicable differentiation condition needs to be explored.

Pig and monkey are among the animals frequently used for drug toxicity test (Dalgaard 2015). In our report, we found that PCOs may be closer to human colonic organoids in drug toxicity response. Our conclusion is in accordance with other studies to indicate that pigs are a good model for drug toxicity test. Firstly, the morphology of pig colon is very similar with human (Kararli 1995). The pH and transit time affecting drug bioavailability in pig colon are also comparable with that of human (Helke and Swindle 2013; Martinez et al. 2002). Secondly, minipigs have widely been accepted as models for toxicity testing of new medicines, especially in Europe (Bode et al. 2010; Ellegaard et al. 2010). Interestingly, the RETHINK project in the Europe has even decided to replace the dogs and non-human primates with minipigs for regulatory toxicity testing (Forster et al. 2010). Thirdly, the activity ratios of cytochrome P450, which mediates drug metabolism and is crucial for xenobiotics biotransformation and toxicity clearance, are closer in minipigs to human, compared to cynomolgus monkey (Dalgaard 2015; Turpeinen et al. 2007).

## Conclusions

In summary, we demonstrated that the expansion medium could support the growth of both porcine and monkey colonic organoids for long-term cultivation. Reduced Wnt signaling and withdrawal of PGE2 induce mature cell differentiation. Furthermore, porcine and human colonic organoids are more similar in the drug sensitivity, suggesting that PCOs could be a better model for the evaluation of anti-colonic cancer drugs.

## Methods

### Animals

Bama miniature pigs used in this study were raised at the Beijing Farm Animal Research Center (affiliated to Institute of Zoology, Chinese Academy of Sciences). The experiments involving pigs were approved by the Animal Ethics Committee of the Institute of Zoology, Chinese Academy of Sciences. The cynomolgus monkey (Macaca fascicularis) colon tissues were obtained from Yunnan Key Laboratory of Primate Biomedical Research, and experimental protocols were approved in advance by the Institutional Animal Care and Use Committee of Yunnan Key Laboratory of Primate Biomedical Research.

### Human colon tissue collection and ethics statement

Human colon tissue was freshly obtained at least 10 cm away from the tumor border in surgically resected specimens at Peking University Third Hospital, Beijing, China, described before (Wang et al. 2020). All samples were obtained with informed consent, and this study was approved by the Peking University Third Hospital Medical Science Research Ethics Committee (M2018083), followed by relevant ethical regulations of Peking University Third Hospital Medical Science Research Ethics Committee.

### Isolation of crypts from porcine, monkey and human colon and organoid culture 

The colon tissue was extracted from three adult male pigs (6 months old) and two cynomolgus monkeys (14 years old) which were euthanized for research. The crypt isolation was conducted based on previous report (Zhao et al. 2015). Briefly, the colon tissue was cut longitudinally and washed by cold PBS for 3–4 times to remove the contaminant and feces. Then, adipose and vascular tissues were removed by operating scalpel. Small pieces of colon tissue (about 10 cm) were incubated in 10 mM EDTA in PBS for 30 min on ice. Next, the pieces were transferred into new PBS and the crypts were released by vigorously scrapping. The epithelial tissue was enriched by centrifugation (3 min at 1,000 rpm). The crypts were then embedded into Matrigel (BD Biosciences) and seeded on 24-well plate. After polymerization, the expansion medium was added. Advanced Dulbecco’s Modified Eagle’s Medium/F12 was supplemented with penicillin/streptomycin, 2 mM GlutaMAX, 1 mM N-acetylcysteine, 1X N2, 1X B-27 to prepare a basal medium (all from Thermo Fisher). The expansion medium was supplemented with 50 ng/mL EGF (Invitrogen), 100 ng/mL Noggin (R&D Systems), 500 ng/mL R-spondin-1 (R&D Systems), 5 μM CHIR-99021 (Selleck), 0.5 μM A-83-01 (Cayman), 10 μM SB202190 (Selleck), 1 nM Gastrin (Tocris), 10 μM Y27632 (Enzo), 2.5 μM PGE2 (Selleck), 10 mM Nicotinamide (Sigma-Aldrich) and 30%Wnt-conditional medium (Wnt3a-CM) (prepared from L-Wnt3a cell line (ATCC)) in basal medium. Growth medium was refreshed every 3–4 days. For passaging at 1:3–1:4 split ratios, the organoids were suspended with 1 ml cold PBS after removal of medium and were pelleted by centrifugation (3 min at 300 g). Then organoids were embedded into new Matrigel in defined medium as indicated in the figure legends. For differentiation experiment, the organoids in the expansion medium were split into new cell plates with the differentiation medium (omission of PGE2 and Wnt3a-CM from the expansion medium, 2.5 μM CHIR-99021) and analyzed after 7 days.

### Immunofluorescence

Immunofluorescence was performed as previously described (Li et al. 2018). Briefly, organoids were fixed in 4% paraformaldehyde for 1 h at room temperature. Organoids were washed by PBS for 3 times and permeabilized by 0.5% Triton X-100 for 1 h at room temperature. Then, samples were blocked with PBT solution (3% BSA and 0.01% Triton X-100 in PBS) for 2 h at room temperature and incubated with primary antibodies overnight at 4 °C. The fluorescein-labeled secondary antibodies (Life Technologies, 1:300) and 4′, 6-diamidino-2-phenylindole (DAPI) were added for 1 h at room temperature next day. The following antibodies were used for immunofluorescence: rabbit anti-Fabp1 (Abcam, ab171739, 1:300); mouse anti-E-cadherin (B&D, 610182, 1:300); rabbit anti-Muc2 (Santa Cruze, sc-15334, 1:300); rabbit anti-ChgA (Abcam, ab15160, 1:300). The images were acquired from Olympus FV3000 Laser Scanning Microscope.

### Alcian blue staining

The colon tissue was fixed with 4% formalin overnight and embedded in paraffin. The sections (5 μm) were de-paraffinized in isopropanol and graded alcohols. Then, sections were stained by Alcian blue staining kit (BASO) according to manufacturer’s instructions. Sections were stained with Alcian blue for 15 min and nuclear fast red for 1 min (BA4087B, Baso). The images were obtained with a Nikon microscope.

### RNA extraction and quantitative RT–PCR

The total RNA was extracted by RNeasy Mini Kit (Qiagen). The cDNA was obtained using Revertra Ace (Toyobo). Then, real-time PCR reactions were performed using qPCR Master Mix (Promega) in triplicates on a LightCycler 480 (Roche). The primers of selected gene were shown in Supplementary Table 1.

### Drug toxicity test

Porcine, monkey and human colonic organoids were seeded into 96 well plates in the differentiation medium with different concentrations of drugs. The concentrations of irinotecan and regorafenib were set based on previous reports (Napolitano et al. 2015; Sim et al. 2018; Yao et al. 2020). After 4 days, organoid growth was monitored with a microscope and cell viability was measured by Cell-Titer Glo 3D Cell Viability Assay (Promega, G9682) according to the manufacturer’s instruction. Cell-Titer reagent (25 μl) was added into each well and incubated with organoids for 30 min at room temperature. Then, the supernatant was collected after centrifugation (500 g, 1 min) and luminescent signals were calculated by luminometer.

### Quantification and statistical analysis

Student’s t-test, one-way or two-way ANOVA analyses were used to compare difference between two groups as indicated in the figure legends. *P < 0.05, **P < 0.01, ***P < 0.001. Data showed in column graphs indicated the mean ± SD. The statistical analysis was carried in GraphPad Prism 6 software. The size of organoids was quantified with Image J. Each experiment was independently repeated at least three times.

## Supplementary Information

**Additional file 1.**

## Data Availability

All data generated or analyzed during this study are included in this published article and the supplementary material. Requests for materials should be addressed to the corresponding author.
